# Human Papillomavirus-Related Cancer Vaccine Strategies

**DOI:** 10.3390/vaccines12111291

**Published:** 2024-11-19

**Authors:** Xia Cai, Ling Xu

**Affiliations:** Department of Gynaecology, Minhang Hospital, Fudan University, Shanghai 201199, China; caicaimoxia1379@163.com

**Keywords:** HPV, vaccine, genital system, head and neck cancer

## Abstract

Background: Human papillomavirus (HPV) persistent infection is a major pathogenic factor for HPV-related cancers, such as cervical cancer (CC), vaginal cancer, vulvar cancer, anal cancer, penile cancer, and head and neck cancer (HNC). Since the introduction of the world’s first prophylactic HPV vaccine, there has been a decline in the incidence of HPV infections and associated cancers. This article reviews the latest literature on the research progress, efficacy, and safety of HPV vaccines for these cancers, providing a reference for HPV vaccination strategy. Methods: By utilizing databases such as PubMed, Google Scholar, CNKI, and Wanfang, we conducted a literature search on research papers related to HPV vaccines from 2014 to 2024, employing keywords such as “HPV”, “HPV vaccine”, “CC”, ”vaginal cancer”, “vulvar cancer”, “anal cancer”, “penile cancer” and “HNC”. Additionally, we reviewed the latest information available on official websites, including the World Health Organization (WHO). Based on the quality and relevance of the papers, we selected over 100 of the most representative articles for further summarization and analysis. Results: Vaccination against HPV can effectively block the transmission of the virus and prevent HPV-related cancers. Current studies have confirmed the efficacy and safety of prophylactic HPV vaccination. However, numerous challenges remain. The global vaccination rate for preventive vaccines remains low, particularly in low- and middle-income countries. Nonetheless, in the future, we can enhance the accessibility, affordability, and coverage of HPV vaccines by expanding the indications of already licensed vaccines, continuously developing new vaccines. Conclusions: The HPV vaccine is an extremely effective measure for the prevention and treatment of HPV-related cancers. Although there are many challenges in expanding the coverage of the HPV vaccine. It is believed that in the not-too-distant future, both prophylactic and therapeutic HPV vaccines will achieve commendable results.

## 1. Introduction

In 1976, Harald Zur Hausen proposed that cervical cancer (CC) is related to infection with human papillomavirus (HPV). Over the following decades, extensive research was conducted on genital HPV, leading to the identification of carcinogenic HPV types [1]. HPV infection can lead to various diseases, including genital warts, respiratory papillomatosis, CC, vaginal cancer, vulvar cancer, anal cancer, penile cancer, and head and neck cancer (HNC) [2,3,4]. By the end of 2023, 143 member states had included HPV vaccine and related services in their national immunization programs, including 13 countries that had newly introduced the vaccine [5]. However, globally, only 15% of young girls have been vaccinated against HPV [6].

Additionally, HPV vaccination programs differ across various countries and regions. Addressing obstacles to vaccination, such as vaccine hesitancy and vaccine availability, along with enhancing the preventive and therapeutic efficacy of HPV vaccines, is an effective approach to improving vaccine coverage and the treatment of HPV-related cancers [7]. This article provides a review of HPV characteristics, HPV vaccines, HPV-related cancers, and the current situation and potential future developments for HPV vaccines.

## 2. Characteristics of HPV

### 2.1. Structural Composition

HPV is a relatively small, non-enveloped icosahedral virus, with a diameter of approximately 52–55 nm [8]. The viral genome is a single double-stranded circular deoxyribonucleic acid (DNA) molecule, around 8 kilobase pairs (kb) in size [8], capable of encoding six early regulatory proteins (E), two late structural proteins (L), and one non-coding upstream regulatory region (URR) [9]. The early regulatory proteins include E1, E2, E4, E5, E6, and E7 [10]. They play important roles in the processes of viral replication, transcription, translation regulation, and cellular transformation, particularly E6 and E7 [10]. The E1 and E2 proteins form a high-affinity hexameric initiation complex that facilitates the unwinding of the double-stranded DNA, thereby initiating viral DNA replication [9]. The E2 protein assists in the integration of viral DNA into the host chromosome [9]. Co-expression of E4 and E1 proteins can halt the cell cycle of differentiated cells at the G2 phase [11]. The E5 protein promotes cell proliferation by activating epidermal growth factor receptor (EGFR) signaling and evades host immunity by inhibiting the formation of major soluble complex molecules, including viral peptides, interferon κ (IFN κ), and growth signaling pathways [12,13].

The p53 protein is an oncogene suppressor in human cells; E6 can lead to the degradation of the p53 protein and can also induce the upregulation of human telomerase reverse transcriptase (hTERT) expression and cell cycle dysregulation [14]. The E7 protein is made up of three conserved regions (CR); CR1 and CR2 are responsible for binding to and degrading retinoblastoma protein (pRb) [15]. The CR3 region affects cell cycle and apoptosis [15]. The late structural proteins include L1 and L2 [10], with their corresponding gene regions occupying nearly 40% of the viral genome downstream of the early region [10]. The L1 protein is the major component of the capsid, forming the viral shell and facilitating the binding of the virus to host cells, while the L2 protein assists in the self-assembly of L1 into the capsid and plays a role during the infection process [16,17]. The URR is believed to be the most variable part of the viral genome, containing binding sites for transcription factors and viral E1 and E2 proteins, which control viral replication and gene expression [2].

### 2.2. HPV Classification

The papillomaviridae family currently encompasses approximately 450 HPV types, with 225 HPV types identified [18]. Based on the homology of the L1 gene nucleotide sequences, HPV is phylogenetically classified into five genera [18]: Alphapapillomavirus (α), Betapapillomavirus (β), Gammapapillomavirus (γ), Mupapillomavirus (μ), and Nupapillomavirus (υ) [18]. Among them, α-HPV (65 types) is isolated from the skin or mucosa of genital and oral lesions and is a cause of many anogenital cancers in humans and primates [19]. The others originate from skin samples, primarily β-HPV (54 types) and γ-HPV (98 types) [19]. α-HPV is further classified into low-risk HPV (LR-HPV) and high-risk HPV (HR-HPV) based on carcinogenic potential [10]. LR-HPV includes HPV 6/11/42/43/44, with HPV 6 and 11 causing benign proliferative diseases in mucosal regions, such as condyloma acuminatum and conjunctival papilloma [10]. HR-HPV includes HPV 16/18/31/33/35/39/45/51/52/56/58/59/68, and approximately 4.5% of global cancers are attributed to persistent HR-HPV infection [2,10].

### 2.3. History of Epidemics

In most cases, HPV infection is transient and asymptomatic [20]. When the skin or mucosa has minor damage, HPV can infect basal layer cells at the point of injury, utilizing the reducing environment of the cell to de-encapsulate and transport DNA into the nucleus, ensuring that the entire lifecycle of HPV remains resistant to cell lysosomal enzymes, thereby reducing clearance by the host [21,22,23]. Due to its unique molecular virological features and immune evasion mechanisms, HPV spreads widely and has a high prevalence among the population [24]. HPV is primarily transmitted through sexual contact, with infection rates largely dependent on the age and sexual behavior of the population [21]. Additionally, it can be transmitted via non-sexual contact and mother-to-child transmission, such as direct skin contact with an infected person sharing personal items, or from a mother to her newborn during childbirth (very rarely) [21].

Approximately 90% of HPV infections are cleared or enter a dormant state within the first one to two years after infection through the host’s humoral and cellular immune responses [25]. After the host is infected with HPV, antibodies are produced, but as time passes, these antibodies may no longer be sufficient to resist the virus, leading to reinfection [26]. Young sexually active women have the highest HPV infection rates, with the peak occurring at about the age of 25 and decreasing thereafter [27]. At around 45 years, a smaller second peak is observed [28,29]. The epidemiology of HPV in men is somewhat different from that in women. Common factors associated with HPV infection in men include human immunodeficiency virus (HIV) infection, number of sexual partners, lack of condom use, race, ethnicity, and circumcision status [7]. A recent meta-analysis revealed that the prevalence of genital diseases associated with HPV among men globally is 31% [30]. HIV-positive men who have sex with men (MSM) are significantly more likely to be infected with multiple genotypes of HPV, with an odds ratio (OR) of 9.30 (95%CI: 3.91–22.1), and they are significantly more likely to carry LR-HPV infections in anal swab samples, with an OR of 6.67 (95%CI: 2.42–18.4), particularly HPV 6, with an OR of 8.92 (95%CI: 3.84–20.7), compared to cervical samples from screening women [31]. The significant impact on quality of life from recurrent anogenital warts (AGWs) and their tendency to progress to invasive anal cancer underscores the urgent need for HPV vaccination. This includes expanding vaccination eligibility to include boys and adults in high-risk populations [31].

## 3. HPV Vaccine

### 3.1. Globally Approved Prophylactic HPV Vaccines

Vaccination against HPV is the most economical and effective means of preventing HPV transmission. HPV vaccines are categorized into prophylactic and therapeutic types, with the former being widely used globally and no therapeutic vaccines currently approved for the market [10,29]. There are two main prophylactic HPV vaccines approved worldwide: Cervarix^®^ from GlaxoSmithKline and Gardasil^®^ from Merck, with Gardasil^®^ available in both 4-valent and 9-valent versions [32,33,34,35] (see Table 1). Moreover, in 2022, the Cervavac^®^ vaccine from the Serum Institute of India received approval for market release within India [36], but it has not been globally promoted.

Cervarix^®^ was licensed by the European Medicines Agency (EMA) in 2007 and by the U.S. Food and Drug Administration (FDA) in 2009, becoming the first cervical cancer vaccine approved in Japan that year [37]. According to GlaxoSmithKline, the vaccine has been approved by 107 regulatory agencies, covering 136 countries worldwide [33]. This indicates Cervarix^®^’s wide acceptance and application globally. Despite its global approval, Cervarix^®^ has been withdrawn from the U.S. market due to low demand [33]. Cervarix^®^ effectively prevents HPV16 and HPV18 infections [34], which cause 70% of cervical cancers [34]. It is suitable for females aged 9–45 [33]. In 2022, the National Medical Products Administration (NMPA) of China approved a two-dose regimen of the Cervarix^®^ vaccine for girls aged 9 to 14 [38].

Gardasil^®^, a 4-valent HPV vaccine, was approved by the FDA and the European Union in 2006. It was the first commercially available HPV vaccine, suitable for females aged 9–45, and prevents diseases caused by HPV infection [39]. Besides HPV16 and 18, Gardasil^®^ also prevents approximately 90% of genital warts caused by HPV6 and 11 infections [40].

Gardasil 9^®^ was licensed by the FDA in 2014 and the European Union in 2015 [37]. It covers five additional HPV types (HPV31/33/45/52/58), providing broader protection [41]. Public information shows that Gardasil 9^®^ has been approved for use in over 70 countries. Initially approved for females aged 9–45 to prevent HPV-related diseases [35], its use has been extended to males in the same age group for preventing HPV-associated diseases such as anal cancer, oropharyngeal cancer, and head and neck cancer [35]. In 2024, NMPA of China approved a two-dose regimen of the Gardasil 9^®^ vaccine for girls aged 9 to 14 [42].

Additionally, since the World Health Organization (WHO) proposed in 2022 to offer alternative single-dose vaccination schedules as an off-label option for individuals aged 9–20, vaccine products originally pre-qualified for a two-dose regimen are increasingly being approved for a single-dose regimen [43]. Global data released on 15 July 2024 show that from 2022 to 2023, the single-dose HPV vaccine coverage rate among girls aged 9–14 increased from 20% to 27% [43]. In 2023, 37 countries implemented single-dose vaccination schedules, covering more than 45% of the 9–14-year-old girls vaccinated that year [43]. As of 10 September 2024, 57 countries had implemented single-dose vaccination schedules [43]. The WHO estimates that at least 6 million more girls were vaccinated against human papillomavirus in 2023 due to the adoption of single-dose schedules [43]. A three-year result from a randomized controlled trial of single-dose HPV vaccine persistence in young women in Kenya found that in the superiority analysis for HPV 16/18, the efficacy of the bivalent and nonavalent vaccines was 97.5% and 98.8%, respectively, while in the superiority analysis for HPV 16/18/31/33/45/52/58, the efficacy of the nonavalent vaccine was 95.5%, indicating that the single-dose HPV vaccine showed high effectiveness, safety, and durable protection [44]. Baisley et al. [45] randomly assigned 930 Tanzanian girls aged 9–14 to receive one, two, or three doses of either the bivalent vaccine or the nonavalent vaccine. They compared the geometric mean concentrations (GMCs) of antibodies and the proportions of seroconversion at two years post-single-dose vaccination with those of 15–20-year-old women randomized to receive a single dose of the same vaccine in the KEN SHE trial. They found that the HPV 16 and HPV 18 antibody GMCs two years after vaccination in the DoRIS trial were similar to or higher than those in the KEN SHE trial, demonstrating that the single-dose HPV vaccine-induced immune responses in the target age group of girls that were likely effective against persistent HPV 16 and HPV 18 infections for at least two years [46]. In September 2023, the UK implemented a single-dose HPV vaccination policy, and research found that this policy not only brought economic benefits but also simplified vaccination management, potentially saving the healthcare system over £1073 million over the next 70 years from switching from a two-dose to a single-dose regimen [46]. While single-dose HPV vaccination can promote vaccine access, health, and gender equity, it is not a panacea, and maintaining and building the capacity to access screening and treatment remain part of the overall HPV strategy [47].

### 3.2. Vaccines Approved in China

Currently, there are five prophylactic HPV vaccines available in China [6,48,49,50]. All these vaccines are administered via intramuscular injection, with the deltoid muscle of the upper arm being the preferred site [51]. Cervarix^®^ received approval from the China Food and Drug Administration (CFDA) in 2016, becoming the first HPV vaccine approved to prevent cervical cancer in China [52]. Gardasil’s 4-valent and 9-valent versions were approved for the Chinese market in 2017 and 2018, respectively [51]. In late August 2022, Gardasil 9^®^ expanded its age range in China, extending its use from females aged 16–26 to those aged 9–45 [51]. In China, the approved HPV vaccines are currently only recommended for use in females and have not been extended for use in males [53]. All vaccination schedules strictly adhere to the instructions provided in the product inserts [53].

Additionally, two domestically produced prophylactic HPV vaccines have been approved for use in China: Cecolin^®^ from Xiamen Innovax Biotech Co., Ltd., Xiamen, China, and WalrinVax^®^ from Yunnan Walvax Biotechnology Co., Ltd., Kunming, China. Cecolin^®^, approved in China on 30 December 2019, is the first HPV vaccine developed independently in China [54]. It is suitable for females aged 9–45 and targets HPV16 and 18, preventing cervical cancer and other related diseases caused by these two types [54]. On 4 October 2024, the WHO announced that Cecolin^®^ could be used with a single-dose vaccination schedule [43]. However, China has not yet approved a single-dose immunization program for HPV vaccines. WalrinVax^®^, approved in China on 22 March 2022, also targets HPV16 and 18, providing more options for cervical cancer prevention [54] (see Table 1). On 2 August 2024, WalrinVax^®^ achieved another significant milestone by receiving WHO prequalification [55]. The vaccine has become the fifth human papillomavirus vaccine on the global market, approved for a two-dose regimen [55].

Since 2021, 11 provinces in China and some cities have included HPV vaccination in government-sponsored public welfare projects, providing free HPV vaccination services for eligible girls aged 13–14 in their regions. As of 18 October 2024, this policy covered approximately 40% of the eligible girls in China [56].

### 3.3. Vaccine Types Under Development Globally: Prophylactic and Therapeutic

All licensed prophylactic HPV vaccines contain virus-like particles (VLPs) formed by the self-assembly of the L1 protein from HPV. Since these vaccines do not include viral genomes, they offer a higher safety profile than live attenuated vaccines [57]. Prophylactic vaccines can induce neutralizing antibodies in the human body, producing a memory effect that protects against HPV infection [10]. The antibody titers generated post-vaccination are ten times higher than those following natural infection, peaking 1 month after the last dose and reaching a plateau 18–24 months later [10].

Therapeutic HPV vaccines activate specific cellular immunity by presenting antigens to immune cells, breaking the immune tolerance of chronic infections, and rebuilding or enhancing the patient’s immune response mechanisms. This helps eliminate pathogens and cancer cells, thereby blocking the spread and metastasis of cancer [58]. Unlike prophylactic HPV vaccines, therapeutic HPV vaccines aim to induce specific cellular immunity rather than humoral immunity (neutralizing antibodies) [59]. Based on the source of tumor antigen expression, therapeutic HPV vaccines can be categorized into four types: nucleic acid vaccines (including DNA and mRNA vaccines), subunit vaccines (including peptide and protein vaccines), recombinant vector vaccines (including bacterial and viral vector vaccines), and dendritic cell vaccines [58]. Currently, the development of new prophylactic and therapeutic HPV vaccines at various clinical stages is summarized in Table 2 [54,59,60].

Certainly, some therapeutic vaccines have achieved notable successes. An Ib/II phase trial evaluating the combination of TG4001 and Avelumab for the treatment of HPV16-positive recurrent/metastatic malignancies showed that among 43 patients, the overall response rate (ORR) was 22% (8/36) for all patients and 32% (8/25) for those without liver metastasis, and it extended the survival duration of the patients (NCT03260023) [61]. A phase II clinical trial assessed the safety and efficacy of ADXS11-001 alone and in combination with cisplatin in patients with recurrent/refractory cervical cancer who had previously undergone chemotherapy and/or radiotherapy. The trial results indicated that ADXS11-001 was generally well-tolerated, with more adverse events reported in the combination treatment. The overall survival (OS) and ORR were similar between the monotherapy and combination therapy groups (OS: 8.28 months vs. 8.78 months; ORR: 17.1% vs. 14.7%), which supports further clinical research [62]. Currently, the ongoing AIM2CERV phase III clinical trial is evaluating the use of ADXS11-001 as adjuvant therapy for high-risk locally advanced cervical cancer after radiochemotherapy to compare the disease-free survival (DFS) of patients receiving ADXS11-001 following platinum-based chemoradiation versus placebo (NCT02853604). PDS0101 is a vaccine composed of the immune-activating cationic lipid R-DOTAP and the HLA-unrestricted HPV16-type E6 peptide. The latest phase II clinical trial is assessing PDS0101 in combination with M9241 (a tumor-targeted immunocytokine consisting of the IL-12 heterodimer) and Bintrafusp alfa (M7824) for patients with HPV16-positive head and neck, cervical, anal, and vaginal cancers. The results showed an ORR of 55.6% among HPV16-positive patients, including two complete responses with tumor shrinkage (NCT04287868).

### 3.4. Vaccine Candidates Under Development in China

China continues to develop new prophylactic HPV vaccines, with over 10 domestically produced HPV vaccines currently in various clinical stages [37,63,64,65] (see Table 3). Due to factors such as the characteristics of HPV, immune evasion, vaccine mechanisms, clinical challenges, and existing treatment methods, there are currently no therapeutic HPV vaccines officially available for use in the population. However, domestic companies have begun researching different therapeutic HPV vaccines [66] (see Table 3). The latest clinical data show that among 35 women with HSIL caused by HPV16 and 18 who were treated with the VGX-3100 vaccine, 91% achieved histological regression six months after treatment completion and avoided clinical surgical excision, with no HPV16 or 18 detected 16 months post-treatment, indicating that VGX-3100 could serve as an alternative to surgery [67]. VGX-3100 is poised to become the first globally developed non-surgical treatment for HPV-associated precancerous lesions (cervical precancer, anal precancer, vulvar precancer, etc.) as well as the first DNA drug of its kind. Among 72 patients with CIN3 treated with the GX-188E DNA vaccine, 52% of the V7 patients (V7 group received the first injection after 20 weeks) and 67% of the V8 patients (V8 group received the first injection after 188 weeks) experienced histological regression following GX-188E injections, demonstrating significant clinical efficacy for treating CIN3 with HPV DNA vaccines [68]. No relevant data have been reported for other Chinese-produced therapeutic vaccines as yet.

## 4. HPV-Related Cancers

HPV infection is a cause of various cancers, including anogenital cancers (cervical, vaginal, vulvar, penile, and anal cancers) and head and neck cancers (oral cavity, oropharyngeal, and laryngeal cancers) [39]. Research indicates that among new cases of cancers, approximately 100% of cervical cancers and anus squamous cell carcinoma, more than 30% of oropharyngeal cancers, about 25% of vulvar and 78% of vaginal cancers, and over 53% of penile cancers are associated with HPV infection [69]. Currently, for HPV-related cancers, apart from cervical cancer, there have been no proposals for implementing different vaccination programs and schedules for various tumor types. However, HPV vaccination programs vary across different countries and regions. The currently administered HPV vaccines cover the major high-risk HPV types, thus addressing HPV-related cancers and have already achieved some interim results.

### 4.1. Cervical Cancer (CC)

CC is the fourth leading cause of cancer-related mortality among women globally [3]. In 2022, there were approximately 661,021 new cases of CC worldwide and about 348,189 deaths, with around 90% of cases occurring in low- and middle-income countries [3]. Progress in reducing the burden of CC in these regions has been slow due to several factors, including the slow expansion of HPV vaccination coverage, low rates of CC screening and early diagnosis, the cost of HPV vaccines, adverse reactions from previous vaccinations for other diseases, and political issues [70,71,72]. In developed countries, there has been a significant decline in the incidence and mortality rates of CC in recent years, attributed to early screening and HPV vaccination among women [39].

A study from Finland regarding the effectiveness of the 2-valent and 4-valent HPV vaccines found that there were no cases of CC or other HPV-related cancers among the vaccinated population, while the incidence rates in the unvaccinated population were 6.4 per 100,000 and 8.0 per 100,000 for CC and other HPV-related cancers, respectively [73]. A retrospective analysis by Steben et al. [74] over the past decade in Canada assessed the impact of the 4-valent HPV vaccine on HPV infection and prevalence, revealing an 86% reduction in the incidence of cervical intraepithelial neoplasia (CIN) among vaccinated individuals. A 2020 study in Sweden showed that women who received at least one dose of the 4-valent HPV vaccine had a 53% lower incidence of CC compared to those who were unvaccinated [75]. A domestic study on HPV vaccination indicated that the efficacy of the HPV vaccine in protecting Chinese women aged 20–45 against persistent cervical HPV infection was 97.5% over 6.5 years [76]. Among women negative for 14 HPV types before vaccination, the incidence of HSIL and cervical surgery related to 9-valent HPV vaccine types was reduced by 98.2% (95% CI: 93.6–99.7) and 97.8% (95%CI: 93.4–99.4), respectively [77]. This highlights the significant impact of HPV vaccination in preventing CC and reducing its incidence, while the continued development of vaccines covering more HPV types and expanding vaccination coverage remains crucial.

### 4.2. Vaginal Cancer

Vaginal cancer is relatively rare, accounting for 1% to 2% of all malignancies of the female reproductive system [78]. Up to 90% of vaginal cancers are associated with high-risk HPV [79]. In 2022, there were 18,800 new cases of vaginal cancer globally, resulting in 8238 deaths [3].

A study by Bertoli et al. [80] that compared the incidence of vaginal cancer before and after the approval of the HPV vaccine in 2006 found a significant decrease of 15.6% in high-grade squamous intraepithelial lesions (HSILs) among women under 30 years old. The incidence of squamous cell carcinoma (SCC) in the vagina fell from 0.5 per 100,000 (1978–1982) to 0.3 per 100,000 (2013–2017). Jacqueline et al. [81] reported that within 17 years following the introduction of the HPV vaccine in the United States, the incidence of vaginal HSIL decreased by 19.1% annually among females aged 15–19. A study from Denmark [82] established a follow-up cohort of 514,537 women aged 17; it compared the incidence of HSIL of the vulva and vagina, revealing an 84% reduction in vaginal HSIL among the 260,571 (50.6%) women who were vaccinated before age 17, compared to unvaccinated women. This was the first observational study to report the effectiveness of HPV vaccination in preventing vaginal HSIL. Since vaginal cancer is less common than vulvar cancer, further research is needed to evaluate the protective effects of HPV vaccination against vaginal cancer.

### 4.3. Vulvar Cancer

Vulvar cancer comprises 2% to 5% of all malignancies of the female reproductive system and is more common in postmenopausal women [83]. In 2022, the global incidence of vulvar cancer was 47,342, with 18,579 deaths [3]. The etiology remains unclear, but it is primarily associated with HPV infection (mainly HPV types 16 and 18), increasing age, immunosuppression, and smoking [83].

Giuliano et al. [77] confirmed in a sample of 10,147 that the 9-valent vaccine reduced the risk of high-grade VIN associated with HPV by 100%. Due to the rarity of high-grade VIN caused by other HPV types, the protective effect of the 9-valent vaccine was predominantly attributed to high-grade VIN caused by HPV type 16, with no statistically significant protection against lesions caused by other types [77]. A study evaluated the effectiveness of HPV vaccination in preventing the recurrence of vulvar HSIL; the results showed that the recurrence rate after surgical treatment for vulvar HSIL was 19% in vaccinated women and 32% in unvaccinated women [84], although the reduction in recurrence rate was not significant. Among young women who were uninfected with HPV, the effectiveness of vaccination in preventing HPV-related infections exceeded 90%, but such pronounced protective effects were not observed in middle-aged women [85]. The protective role of HPV vaccination against vulvar cancer is well supported by evidence, indicating that vaccination can reduce the risk of vulvar lesions by over 90% for women without prior HPV infection, thus contributing to the prevention of vulvar cancer.

### 4.4. Anal Cancer

In recent years, the incidence of anal cancer worldwide has been increasing, particularly among men who have sex with men (MSM), HIV-infected individuals, HPV-infected individuals, and patients with gynecological cancers [7]. In 2022, the global incidence of anal cancer was 54,194 cases, with 21,960 deaths [3]. Anal cancer is primarily caused by HPV infection (80%), with HPV16 being the most common type [86]. The HPV infection and transformation patterns in anal cancer are similar to those in cervical cancer (CC) [87]. Most anal cancers are SCC, occurring mainly in the anal canal and surrounding areas [88]. Men who have sex with men living with HIV (MSM LWH) are at the highest risk of HPV-related anal cancer [89]. A study assessing the prevalence and severity of anal HPV disease among MSM LWH under the age of 35 conducted anorectal cytology screening on 1255 men who have sex with men aged 18–34 from 2014 to 2020. Of these, 916 underwent HR-HPV co-testing, and 467 received high-resolution anoscopy (HRA) and biopsies. The findings revealed that 19% had received at least one dose of the HPV vaccine, with a cytological abnormality rate of 65% and HR-HPV and HPV16 prevalence rates of 87% and 30%, respectively [89]. The biopsy results showed benign conditions (10%), LSIL (43%), and HSIL (47%), with no prevalent or sporadic cases of anal cancer reported. Despite the high prevalence of anal HR-HPV infection and precancerous lesions, no cases of anal cancer were found in a large cohort of MSM aged under 35 [89]. In a global multi-center quadrivalent HPV vaccine trial placebo group, the incidence rates of incident persistence (IP) anogenital HPV infections were assessed in 295 MSM and 1576 heterosexual men (HM) aged 16–27. The IP infection rates per 100 person-years for the four- and nine-valent HPV types in HM were 4.1 (95%CI: 3.5–4.9) and 6.8 (95%CI: 5.9–7.6) for the penis/scrotum, and 1.2 (95%CI: 0.8–1.6) and 1.9 (95%CI: 1.5–2.4) for the perineum/perianal area; for MSM, the rates were 2.3 (95%CI:1.3–3.8) and 3.2 (95%CI:2.0–4.9) for the genital/scrotum, 6.8 (95%CI: 4.9–9.2) and 9.0 (95%CI: 6.9–11.6) for the perineum/perianal area, and 12.0 (95%CI: 9.4–15.1) and 16.8 (95%CI: 13.7–20.2) for the anus, respectively. The cumulative IP incidence rates over 36 months (excluding the anal canal and any 9vHPV type) were higher in MSM than in HM (24.1% vs. 18.4%) [90]. A randomized controlled study involving 237 MSM aged 16–26 found that the incidence of anal intraepithelial neoplasia (AIN) or anal cancer in those vaccinated with the quadrivalent HPV vaccine was only 2.0 per 100,000 over seven years, compared to 90.6 per 100,000 in the placebo group [91]. Additionally, the effectiveness of at least one dose of the HPV vaccine in preventing anal HPV infections was 59% for males aged ≤ 18 and 18% for males aged > 18 [92], indicating that males should receive the HPV vaccine at an early age.

### 4.5. Penile Cancer

Penile cancer is extremely rare but has a high disability rate [93]. In 2022, there were 37,699 new cases of penile cancer globally, with 13,729 deaths [3]. HPV infection is considered a significant risk factor for penile cancer, with HPV16 being the most common subtype [94]. Bai et al. found that among 300 HIV-positive MSM, 600 HIV-negative MSM, and 659 MSW, the infection rates for HPV genotypes covered by the nonavalent vaccine were 47.0%, 36.8%, and 3.5%, respectively, and the co-infection rates for the anus and genitals were 20.3%, 14.2%, and 0.6%, respectively, while the co-infection rates for the anus, genitals, and mouth were 1.3%, 0.3%, and 0, respectively [95]. Also, 77.0% of HIV-positive MSM and 75.3% of HIV-negative MSM indicated willingness to receive the HPV vaccine, whereas only 58.9% of MSW expressed unwillingness [95]. A cross-sectional study of 687 MSM tested for HPV in penile swab specimens compared the HPV infection rates between vaccinated and unvaccinated individuals. The results showed that the HPV infection rate in MSM vaccinated before the age of 18 was significantly lower than that in unvaccinated individuals, with an efficacy of 85% in preventing quadrivalent HPV-related infections [96]. Further clinical trials with longer observation periods or larger sample sizes are needed to assess the protective effect of the vaccine against penile cancer.

### 4.6. Head and Neck Cancer (HNC)

In HNC, HPV has the highest attributable risk for oropharyngeal cancer, while the association with other cancer types is relatively low [10]. In 2022, there were 946,456 new cases of HNC and 482,001 deaths [3]. Approximately 90% of these cases are head and neck squamous cell carcinoma (HNSCC) [3]. The incidence of HNSCC has been rising primarily due to HPV infection, particularly HPV16 and HPV18 [97]. Additionally, tobacco use, chronic alcohol consumption, mechanical irritants, radiation exposure, and various occupational exposures contribute to the increased incidence of HNC [98]. Epstein-Barr virus and hepatitis B virus are other pathogenic factors for HNC, with Epstein-Barr virus considered a biomarker for nasopharyngeal carcinoma [99,100].

A study by Chaturvedi et al. [101] involving 8067 participants in the National Health and Nutrition Examination Survey (NHANES) from 2011 to 2014 found that four years after vaccination, the oral HPV 6/11/16/18 infection rate was significantly lower in the HPV vaccine group compared to the unvaccinated group, especially among men. Schlecht et al. [102] reported that the oral HPV 6/11/16/18 infection rate was reduced by 83% in adolescent females who received more than one dose of the quadrivalent vaccine. Research on 1784 high school students aged 14–17 in Colombia [103] found that the oral HPV16 infection rate was reduced by 72% in those who received two doses of the quadrivalent HPV vaccine. Notably, the risk of oral HPV16 infection was higher in males, with the vaccinated group consisting entirely of females and 88.7% of the unvaccinated group being male [103]. Chaturvedi et al. [104] suggested that the HPV vaccine may provide herd immunity, with the decrease in oral HPV infections among males potentially attributed to increased vaccination uptake among females, while the lack of significant herd immunity for females may be due to the low prevalence of oral HPV infections in women.

Modeling studies on HPV vaccination among males in the United States indicated that, at the current vaccination rate, if coverage were to increase to 80%, the incidence of HPV-related oropharyngeal cancer is expected to decline significantly after 2060 [105]. We believe that with the implementation of HPV vaccination, reductions in smoking rates, promotion of safe sexual practices, and early accurate diagnoses, the incidence of HNC will significantly decrease in the future.

## 5. The Present and Future of HPV Vaccines

### 5.1. HPV Vaccination Status and Vaccination Recommendations

In 2018, the World Health Organization (WHO) issued a global call to action for the elimination of CC [106]. This initiative aims to achieve the 90-70-70 targets by 2030, which are defined as 90% of girls fully vaccinated against HPV by the age of 15, 70% of women receiving two high-efficiency screenings in their lifetime at the ages of 35 and 45, and 90% of women diagnosed with cervical disease receiving treatment and care [106]. The success of this initiative relies on high vaccination rates, widespread screening, and ensuring timely treatment, thus overlooking many barriers to women’s access to healthcare in various regions, including economic hardship, cultural stigma, and inadequate infrastructure [107]. However, due to various reasons such as insufficient awareness of the importance of HPV infection and vaccination, the role of schools in improving HPV acceptance not being emphasized, insufficient participation of healthcare professionals in HPV vaccination, misinformation, cultural resistance, and the cost of vaccination, parents often exhibit vaccine hesitancy, whether for themselves or their children, which further affects the promotion and coverage of vaccination efforts [72,107]. As of now, only 15% of young girls globally have received the preventive HPV vaccine [6]. Although the HPV vaccine has successfully prevented certain types of HPV infections, it is most effective when administered before individuals become sexually active and are exposed to the virus [108].

The Advisory Committee on Immunization Practices (ACIP) of the Centers for Disease Control and Prevention (CDC) approved the quadrivalent HPV vaccine for the first time in 2006, recommending it for administration at ages 11 to 12, with the earliest possible age being 9 [109]. For those who have not been adequately vaccinated before, the age limit can be extended up to 26 [110]. In 2015, ACIP recommended the nine-valent vaccine as one of the three HPV vaccines for routine vaccination, advising vaccination for females aged 13 to 26 and males aged 13 to 21 who have not yet been vaccinated [111,112]. The quadrivalent HPV vaccine was approved in Canada in 2006 for use in females aged 9 to 26, and in 2010, it was approved for males aged 9 to 26; in 2011, the vaccination age was further expanded to include females under 45 [113,114]. The bivalent HPV vaccine was approved for Canadian women in 2010, and the nine-valent HPV vaccine was approved for both males and females in 2015 [115]. Some cities or regions may provide partial government funding, but the primary mode of vaccination remains out-of-pocket payment [116]. Although vaccinating females against HPV can benefit most men who have sex with women (MSW), it does not provide the same protection for men who have sex with men (MSM) [116]. Research indicates that the HPV infection rate among MSM is significantly higher than that among MSW [117,118]. Additionally, the incidence rate of anal cancer among MSM is comparable to that of individuals who have never received the HPV vaccine (80 per 100,000 per year) [119]. Therefore, in promoting HPV vaccination and developing vaccination programs, consideration should be given to extending this protection to the MSM population.

Due to the relatively low cost-effectiveness of promoting the HPV vaccine among males, only a few developed countries, such as Australia and the United Kingdom, offer free HPV vaccination for males. Certainly, significant achievements have been made as well. The Australian government-funded HPV vaccination program was launched in April 2007, targeting girls and young women, and was expanded to include boys in February 2013. As of now, all females born in Australia under the age of 38 and males under the age of 21 are eligible for free vaccination with either the quadrivalent or nonavalent HPV vaccine [120]. The latest data show that in 2021, 80.3% of girls completed the full HPV vaccine course before the age of 15, and for boys, the figure reached 77.2% [121]. The hospitalization rates for genital warts in both women and men have been gradually decreasing, with a more pronounced reduction of 58% for women and 45% for MSW [122]. In the UK, government-funded HPV vaccination was introduced in September 2008, initially targeting girls aged 12–13 with the bivalent HPV vaccine. In September 2012, the vaccine was switched to the quadrivalent HPV vaccine, and in September 2019, boys were also included in the vaccination program. Subsequently, the scope was expanded to include females and males aged 13–45 who had not been vaccinated against HPV [123]. In 2018, the first-dose HPV vaccination rate among 12–13-year-old girls in the UK reached 89%, and in 2020, the first-dose vaccination rate among 12–13-year-old boys reached 85% [114]. The successful implementation of the HPV vaccination program has significantly reduced the incidence of HPV-related diseases. For example, the incidence of cervical pre-cancerous lesions has decreased significantly among those vaccinated with the HPV vaccine. The extension of HPV vaccination to males will further reduce the incidence of HPV-related diseases, such as anal cancer, oral cancer, and genital warts. It is evident that a government-led comprehensive promotion of HPV vaccination, such as through public health campaigns and educational programs, can greatly enhance vaccination rates and ensure the standardization and timeliness of the vaccination process. Additionally, there is a need to lower vaccination costs, expedite the vaccine delivery system, and strengthen the monitoring of vaccination data to ensure the smooth process of vaccination [107].

### 5.2. Cost-Effectiveness of Vaccination

The cost-effectiveness of HPV vaccination can influence the coverage of vaccination, especially in low- and middle-income countries [72]. According to the adjusted cost per dose of HPV vaccine delivery in 2022 (excluding the cost of vaccine procurement), Sri Lanka’s routine school-based HPV vaccination program has the lowest financial cost per dose (at USD 0.31), while Zimbabwe’s school demonstration program has the highest financial cost per dose (at USD 24.70) [124]. Within the same country, cost differences exist due to geographical location or distribution settings. For example, in Mozambique, the economic cost per fully immunized girl (FIG) (including HPV vaccine procurement costs) in one region is more than double the same unit cost in the other three regions [125].

With the development of vaccines, GSK’s bivalent HPV vaccine is USD 10.25–14.14 lower per dose than Meck’s quadrivalent HPV vaccine [72]. Over the past five years, prices have generally declined across all procurement and income groups. If there is reasonable demand for these new products, the emergence of future new vaccines will create a more competitive environment, leading to further price reductions [72]. A study in the United States found that, despite the high cost per dose of the nonavalent HPV vaccine, it was cost-effective at all coverage levels and beneficial for health outcomes, especially in states with low vaccination rates [126]. Similarly, Thai researchers found that compared to no vaccination, all vaccination programs achieved between 41,298–71,057 quality-adjusted life years (QALYs) and saved between USD 14,914,186 and USD 19,821,655 in costs [127]. The strategy of administering two doses of the nonavalent HPV vaccine was rated as the most cost-effective, showing a cost-effectiveness of USD 406 per QALY, within the range of lower willingness-to-pay thresholds [127]. Domestic research in China found that various combined screening and HPV vaccination strategies would generate an additional USD 6,157,000–22,146,000 compared to no intervention, but the most cost-effective cervical cancer prevention strategy in China is to perform cervical cancer HPV screening every five years and vaccinate [54].

### 5.3. Efficacy and Safety of HPV Vaccine

The efficacy of the HPV vaccine refers to the ability of the body to prevent HPV infection and related cancers following vaccination. In a multinational, double-blind, placebo-controlled trial, 12,000 women aged 15–26 who received the quadrivalent HPV vaccine showed a 98% protective rate against CIN2 and CIN3+, HPV16 and HPV18 infections, and adenocarcinoma in situ (AIS) [119]. A 2019 study found that antibodies induced by the nonavalent vaccine could cross the placenta, thereby protecting the fetus from HPV6 and HPV11 infections [128]. A recent study in England assessed the national HPV vaccination program launched in 2008 for girls aged 12–13, showing that vaccinated females had an 83.9% reduction in CC incidence and a 94.3% reduction in CIN3 incidence compared to unvaccinated women [114]. A study of the long-term immunogenicity of the quadrivalent vaccine revealed sustained antibody responses to HPV 6/11/16/18 for up to 14 years post-immunization [127]. These studies indicate that the current vaccines are effective, with antibodies maintained for an extended period, significantly reducing the risk of HPV-related cancers.

HPV vaccines exhibit good safety profiles, although they come with some post-vaccination adverse reactions, most of which are mild and of short duration. The most common reactions include redness and pain at the injection site [129]. The bivalent vaccine tends to cause headaches, fever, vomiting, dizziness, muscle pain, and diarrhea [130]. A cohort study of Danish and Swedish girls receiving the quadrivalent HPV vaccine did not find connections to neurological, immune, or thromboembolic side effects [131]. The nonavalent vaccine, using aluminum as an adjuvant and with virus-like particles more than twice the size of the quadrivalent vaccine, showed more systemic and local adverse reactions [132]. Concerns have been raised about a potential link between HPV vaccination and autoimmune diseases [133]. However, a large-scale study involving 3,983,824 women (including 789,082 who received the quadrivalent HPV vaccine) found no significant association between the vaccine and multiple sclerosis or other demyelinating diseases [134]. HPV vaccines are currently regarded as safe, effectively preventing vaccine-type HPV infections and associated cellular abnormalities, including pre-cancerous and benign lesions [135].

### 5.4. Long-Term Effects and Follow-Up Studies

Long-term follow-up studies are also needed to evaluate the enduring immunological effects and long-term safety of vaccines. Monitoring vaccine efficacy includes assessing the impact of vaccination on the incidence of HPV-related cancers and the resulting population-level immunity. A retrospective study in Scotland examining the impact of bivalent HPV vaccination at age 12–13 on cervical disease in women aged 20 found that, compared to unvaccinated women born in 1988, vaccinated women born in 1995 and 1996 experienced an 89% reduction (95%CI: 81%–94%) in the incidence of CIN 3+ lesions, an 88% reduction (95%CI 83%–92%) in CIN 2+ lesions, and a 79% reduction (95%CI:69%-86%) in CIN 1 lesion s [136]. The younger the age at vaccination, the more significant the vaccine’s effect: women vaccinated at 12–13 years old showed an 86% reduction (95%CI:75%-92%) in CIN 3+, while women vaccinated at 17 years old showed a 51% reduction (95%CI: 28%–66%) [136]. Furthermore, among women who received at least one dose of the bivalent HPV vaccine at 12–13 years old, no cases of invasive cervical cancer were observed [137].

Recent data from England indicate that women who receive HPV vaccination as part of a routine program show higher vaccine effectiveness, with reductions of 89% (95%CI: 81–94%) for CIN 3, 88% (95%CI: 83–92%) for CIN 2, and 79% (95%CI: 69–86%) for CIN 1 [136]. Wen et al.’s study on Chinese women aged 20–45 who were vaccinated with the quadrivalent HPV vaccine found that the vaccine maintained its immunogenicity and protective effect against precancerous cervical lesions over the past 13 years [138]. A study from the Netherlands compared and evaluated HPV16 variants in the URR, L1, and E6 genes among young women who were and were not vaccinated against HPV, finding moderate HPV16 sequence diversity. HPV16-positive viral loads were detected in 1.9% (17/875) of vaccinated women and 13% (162/760) of unvaccinated women, demonstrating the high efficacy of the vaccine [139]. Since the introduction of HPV vaccination in Germany in 2007, Grieger et al.’s [140] latest study found that 11 years after the introduction of HPV vaccination in Germany, a decline in the incidence of cervical cancer at the population level could be observed among birth cohorts eligible for vaccination. For example, between 2010 and 2018, the incidence rate among women aged 24–26 decreased from 70.0 cases per 100,000 people annually to 41.8 cases.

Research into the co-administration of HPV vaccines with other vaccines, such as those for measles, rubella, and influenza, should be conducted to optimize vaccination schedules and enhance coverage and efficiency. Through these efforts, the effectiveness and impact of HPV vaccines will be further enhanced, contributing to the prevention and treatment of HPV-related diseases, particularly cancers.

### 5.5. Future Efforts for HPV Vaccines

Expanding the scope of approved HPV vaccines to cover high-risk populations, such as transplant recipients, HIV-infected individuals, and men who have sex with men, is a key focus [141], as well as developing more diverse types of HPV vaccines, which are broader-spectrum and can protect against more HPV types [141]. Additionally, creating vaccines that can treat existing HPV infections, particularly for patients with early-stage cancers or precancerous lesions, would provide substantial benefits.

Currently, the potential mechanisms of HPV immune evasion in cancers remain unclear. Future research could focus on elucidating the effects of HPV infection on the local tissue microenvironment, exploring the relationships between HPV and other immune checkpoints, assessing the potential of immune checkpoint blockade strategies in HPV-related cancers, delving into the mechanisms of HPV integration into the host genome and its impact on genomic instability and mutation accumulation, and investigating the relationships between HPV and cytokine balance and their effects on immune responses [4].

## 6. Conclusions

HPV, owing to its unique molecular virology characteristics and immune evasion mechanisms, boasts a remarkably high infection rate among the population. Vaccination against HPV can effectively block the transmission of the virus and prevent HPV-related cancers. Current studies have confirmed the efficacy and safety of prophylactic HPV vaccination in preventing HPV infections and associated cancers. However, numerous challenges remain. Under the influence of economic conditions, healthcare resources, educational levels, and awareness, the global vaccination rate for preventive vaccines remains low, particularly in low- and middle-income countries. Nonetheless, in the future, we can enhance the accessibility, affordability, and coverage of HPV vaccines by expanding the indications of already licensed vaccines, continuously developing new vaccines, and conducting in-depth research on the mechanisms of HPV immune evasion in cancers.

## Figures and Tables

**Table 1:** Prophylactic HPV vaccines that have been approved for marketing.

	Prophylactic HPV Vaccines Approved Globally			Prophylactic HPV Vaccines Produced and Approved in China	
Valency (Brand name /manufacturer)	2vHPV (Cervarix^®^/GlaxoSmithKline)	4vHPV (Gardasil^®^/Merck)	9vHPV (Gardasil9^®^/Merck)	2vHPV (Cecolin^®^/Innovax Biotech)	2vHPV (WalrinVax^®^/Zerun biotech)
Component content	Per dose contains HPV 16 L1 protein 20 µg, HPV 18 L1 protein 20 µg	Per dose contains HPV 6 L1 protein 20 µg, HPV 11 L1 protein 40 µg, HPV 16 L1 protein 40 µg, HPV 18 L1 protein 20 µg.	Per dose contains HPV 6 L1 protein 30 µg, HPV 11 L1 protein 40 µg, HPV 16 L1 protein 40 µg, HPV 18 L1 protein 40 µg, and HPV 31/33/45/52/58 L1 proteins each 20 µg.	Per dose contains HPV 16 L1 protein 40 µg, HPV 18 L1 protein 20 µg	Per dose contains HPV 16 L1 protein 40 µg, HPV 18 L1 protein 20 µg
Expression system	Baculovirus	Saccharomyces cerevisiae	Saccharomyces cerevisiae	Escherichia coli	Pichia pastoris
Target population	Women aged 9–45	Women and men aged 9–45	Women and men aged 9–45	Women aged 9–45	Women aged 9–30
Immunisation schedule	Three doses at months 0, 1, and 6 (for girls aged 9–14, two doses at months 0 and 6), each dose being 0.5 mL	Three doses at months 0, 2, and 6, each dose being 0.5 mL	Three doses at months 0, 2, and 6 (for girls aged 9–14, two doses at months 0 and 6), each dose being 0.5 mL	Three doses at months 0, 1, and 6 (for girls aged 9–14, two doses at months 0 and 6), each dose being 0.5 mL	Three doses at months 0, 2, and 6 (for girls aged 9–14, two doses at months 0 and 6), each dose being 0.5 mL
Adjuvant	AS04 [containing 3-O-desacyl-4’-monophosphoryl lipid A, aluminium hydroxide]	Aluminium phosphate sulphate	Aluminium phosphate sulphate	Aluminium hydroxide	Aluminium phosphate
Ingredient	Sodium chloride, disodium dihydrogen phosphate dihydrate, water for injection	Aluminium (amorphous aluminium hydroxyphosphate sulphate adjuvant), sodium chloride, L-histidine, polysorbate 80, sodium borate, water for injection	Aluminium (amorphous aluminium hydroxyphosphate sulphate adjuvant), sodium chloride, L-histidine, polysorbate 80, sodium borate, water for injection	Sodium chloride, disodium dihydrogen phosphate dihydrate, sodium hydrogen phosphate dihydrate, polysorbate 80, water for injection	Sodium chloride, histidine, polysorbate 80, water for injection
Prevented diseases	Cervical pre-cancerous lesions and cervical cancer caused by HPV16/18 infection	Pre-cancerous lesions, cancers, and genital warts caused by HPV6/11/16/18 infection	Pre-cancerous lesions, cancers, and genital warts caused by HPV infection types 6, 11, 16, 18, 31, 33, 45, 52, and 58	Cervical pre-cancerous lesions and cervical cancer caused by HPV16/18 infection	Cervical pre-cancerous lesions and cervical cancer caused by HPV16/18 infection
Global/China launch time (year)	2007/2016	2006/2017	2014/2018	-/2019	-/2022

Note: HPV, human papillomavirus.

**Table 2:** Globally in-clinical-stage prophylactic and therapeutic HPV vaccines.

Type	Name	Research and Development Stage	Antigen Information	Research and Development Unit
Prophylactic	AAVLP-HPV	Phase I	L1 protein of HPV 16 and HPV 18	CureVac AG, Tubingen, Germany
	PANHPVAX	Early Phase I	L1 protein of HPV 16 and HPV 18	Panacea Biotec, New Delhi, Indian
	VA-HPV	Phase II	L1 protein of HPV 16 and HPV 18	Vaxart, Inc., San Francisco, CA, USA
	TheraVax	Some of the studies are in phase I/II clinical trials	The L1 and E7 proteins of HPV	TheraVax, Inc., Sao Paulo, Brazil
	MVA-HPV	Phase II	L1 protein of HPV 16 and HPV 18	Multiple universities and research institutes worldwide
Therapeutic	VGX-3100	Phase III	Two plasmids encoding E6 and E7 protein of HPV16 and 18	Inovio Pharmaceuticals, Plymouth Meeting, PA, USA
	INO-3112	Phase II	It contains two plasmids, VGX-3100 and INO-9012, INO-9012 expresses IL-12	Inovio Pharmaceuticals
	MVA-E2	Phase III	The core antigen is the E2 protein of HPV	The University of Oxford and its partners
	BNT113	Phase II	HPV L1 protein and HPV E7 protein	BioNTech, Mainz, Germany
	ChAdOx1-HPV + MVA-HPV	Phase II	HPV E6 protein and HPV E7 protein	The University of Oxford and its partners
	Ad26. HPV16/18	Phase II	The E7 protein of HPV types 16 and 18	Janssen Pharmaceuticals, Berce, Belgium
	SGN-00101	Phase II	HPV E6 and E7 proteins	ADC Therapeutics S.A., Lausanne, Switzerland
	GX-188E	Phase II	HPV E6 and E7 proteins	Genexine, Inc., Seongnam, Republic of Korea
	GENUINE	Phase II	The E6 and E7 proteins of HPV types 16 and 18	Merck & Co., Rahway, NJ, USA
	TRINITY	Phase II	HPV E6 and E7 proteins	Inovio Pharmaceuticals
Therapeutic	ISA101b	Phase II	HPV type 16 E7 protein	ISA Pharmaceuticals, Uhrstheist, The Netherlands
	PDS0101	Phase II	The E6 and E7 proteins of HPV types 16 and 18	PDS Biotechnology, Princeton, NJ, USA
	HB-201	Phase II	The E6 and E7 proteins of HPV type 16	Gen009
	HB-202	Phase II	The E6 and E7 proteins of HPV types 16 and 18	Gen009
	DPX-E7 vaccine	Phase II	E7 protein of HPV type 16	ImmuNext, Lebanon, NH, USA
	ADXS11-001	Phase II	The E7 protein of HPV types 16 and 18	Advaxis, Inc., Princeton, NJ, USA
	TG4001	Phase II	The E6 and E7 proteins of HPV type 16	Transgene, Boston, MA, USA
	TA-CIN Vaccine	Phase I	L1 protein of HPV	Transgene
	pNGVL4aCRTE6E7L2	Phase I	The E6 and E7 proteins of HPV type 16	Butantan Institute
	P16-37-63	Phase I	p16 protein, the E6 and E7 proteins of HPV type 16	CureVac, Tubingen, Germany
	GTL001	Phase I	HPV E6 and E7 proteins	GlaxoSmithKline, Hong Kong, China
	GL-0810	Phase I	HPV 16 E6 and E7 proteins	Genexine, Inc.
	VB10.16	Phase II	L1 proteins of HPV 16 and 18	VBI Vaccines, Inc., Cambridge, MA, USA
	pBI-1101	Phase II	HPV 16 E6 and E7 proteins	Beijing Institute of Biological Products (BIBP), Beijing, China
	NWRD08	Phase I	E6 and E7 proteins of HPV16 and 18	Novartis, Hong Kong, China

Note: HPV, human papillomavirus; INC, incorporated.

**Table 3:** In-clinical-phase prophylactic and therapeutic HPV vaccines in China.

Type	Name	Development Clinical Phase	Registration Number	Development Unit
Prophylactic	Recombinant bivalent HPV (types 16/18) vaccine (Hanseniaspora yeast)	Phase I has been completed	CTR20182556	Jiangsu Recbio Technology Co., Ltd., Taizhou, China
	Recombinant bivalent HPV (types 6/11) vaccine (Hanseniaspora yeast)	Phase I has been completed	CTR20210109	Jiangsu Recbio Technology Co., Ltd.
	Recombinant trivalent HPV (types 16/18/58) vaccine (Escherichia coli)	Phase III	CTR20201795	Beijing Health Guard Biotechnology Inc., Beijing, China
	Recombinant quadrivalent HPV (types 6/11/16/18) vaccine (Hanseniaspora yeast)	Phase III	CTR20221050	Shanghai Bovax Biotechnology Co., Ltd., Shanghai, China
	Recombinant quadrivalent HPV (types 16/18/52/58) vaccine (Pichia pastoris)	Phase II has been completed	CTR20190482	Shanghai Institute of Biological Products Co., Ltd., Shanghai, China
	Recombinant quadrivalent HPV (types 6/11/16/18) vaccine (Hanseniaspora yeast)	Phase III	CTR20171662	Chengdu Institute of Biological Products Co., Ltd., Chengdu, China
	Recombinant quadrivalent HPV (types 6/11/16/18) vaccine (Hanseniaspora yeast)	Phase III has been completed	CTR20171662	Chengdu Institute of Biological Products Co., Ltd./Beijing Institute of Biological Products Co., Ltd., Beijing, China
Prophylactic	Recombinant nine-valent HPV (types 6/11/16/18/31/33/45/52/58) vaccine (Escherichia coli)	Phase III	CTR20210947	Jiangsu Recbio Technology Co., Ltd.
	Recombinant nine-valent HPV (types 6/11/16/18/31/33/45/52/58) vaccine (Escherichia coli)	Phase III (women aged 9–26 years)	CTR20220679	Beijing Health Guard Biotechnology Inc.
	Recombinant nine-valent HPV (types 6/11/16/18/31/33/45/52/58) vaccine (Escherichia coli)	Recruitment completed (Chinese male participants)	CTR20223306	Beijing Health Guard Biotechnology Inc.
	Recombinant nine-valent HPV (types 6/11/16/18/31/33/45/52/58) vaccine (Escherichia coli)	Application for market authorization submitted	CXSS2400088 and CXSS2400089	Xiamen Innovax Biotech Co., Ltd., Xiamen, China/Xiamen University
	Recombinant nine-valent HPV (types 6/11/16/18/31/33/45/52/58) vaccine (Hanseniaspora yeast)	Phase III	CTR20242197	Shanghai Bovax Biotechnology Co., Ltd.
	Recombinant nine-valent HPV (types 6/11/16/18/31/33/45/52/58) vaccine (Pichia pastoris)	Phase III	CTR20200365	Shanghai Zerun Biotechnology Co., Ltd., Shanghai, China
	Recombinant 11-valent HPV (types 6/11/16/18/31/33/35/39/45/52/58) vaccine (Hanseniaspora yeast)	Phase III	CTR20221258	Sinopharm Zhongsheng Biotechnology Research Institute Co., Ltd., Shanghai, China/Beijing Institute of Biological Products Co., Ltd./Chengdu Institute of Biological Products Co., Ltd.
	Recombinant 14-valent HPV (types 6/11/16/18/31/33/35/39/45/51/52/56/58/59) vaccine (Insect cell)	Phase II	SCT1000	Sinocelltech Group Ltd., Beijing, China
	Recombinant 15-valent HPV (types (6/11/16/18/31/33/35/39/45/51/52/56/58/59/68) vaccine (Escherichia coli)	Phase I	CTR20241041	Liaoning Chengda Biotech Co., Ltd., Shenyang, China/Beijing Health Guard Biotechnology Inc.
Prophylactic	Recombinant 15-valent HPV (types 6/11/16/18/31/33/35/39/45/51/52/56/58/59/68) vaccine (Hanseniaspora yeast)	Phase I	CTR20242006	Shanghai Bovax Biotechnology Co., Ltd.
Therapeutic	VGX-3100	Phase III	CTR20201547	Inovio Pharmaceuticals, Inc./Alliance Medical Products Inc., Irvine, CA, USA/Beijing Apollo Saturn Biomedical Technology Co., Ltd., Beijing, China
	NWRD08	Phase I	CTR20240040	Newish Biotechnology (Wuxi) Co., Ltd., Nanjing, China
	AFN0328	Clinical trial approval	CXSL2400268 and CXSL2400288	Hefei Afana Biotechnology Co., Ltd., Hefei, China/Anhui Anke Biotechnology (Group) Co., Ltd., Hefei, China
	LY01620	Phase I	CTR20243545	Nanjing Geneleap Biotech Co., Ltd., Nanjing, China
	SYS6026	Clinical trial application	CXSL2300296 and CXSL2300297	CSPC Megalith Biopharmaceutial Co., Ltd., Shijiazhuang, China
	ARC01	Phase I	CXSL2300755	Nanjing Auro Biotechnology Co., Ltd., Nanjing, China

Note: HPV, human papillomavirus; Co., Ltd., company limited; Inc., incorporated; CTR, clinical trial registry; CXSS, clinical study summary synopsis; CXSL, clinical study summary letter; SCT, stem cell therapy (the registration number is not for the vaccine itself but for the trial in which the vaccine is being assessed).
